# An uncommon case of random fire-setting behavior associated with Todd paralysis: A case report

**DOI:** 10.1186/1471-244X-12-132

**Published:** 2012-08-31

**Authors:** Masayuki Kanehisa, Katsuhiko Morinaga, Hisae Kohno, Yoshihiro Maruyama, Taiga Ninomiya, Yoshinobu Ishitobi, Yoshihiro Tanaka, Jusen Tsuru, Hiroaki Hanada, Tomoya Yoshikawa, Jotaro Akiyoshi

**Affiliations:** 1 Department of Neuropsychiatry, Oita University Faculty of Medicine, Oita, Hasama-Machi, 879-5593, Japan; 2 FUJIFILM RI Pharma Co., Ltd., Tokyo, 104-0031, Japan

**Keywords:** Fire setting, Arson, Lacunar stroke, Frontal lobe dysfunction, Focal epilepsy, Prolonged seizures, Ictal paralysis, Todd’s paresis

## Abstract

**Background:**

The association between fire-setting behavior and psychiatric or medical disorders remains poorly understood. Although a link between fire-setting behavior and various organic brain disorders has been established, associations between fire setting and focal brain lesions have not yet been reported. Here, we describe the case of a 24-year-old first time arsonist who suffered Todd’s paralysis prior to the onset of a bizarre and random fire-setting behavior.

**Case presentation:**

A case of a 24-year-old man with a sudden onset of a bizarre and random fire-setting behavior is reported. The man, who had been arrested on felony arson charges, complained of difficulties concentrating and of recent memory disturbances with leg weakness. A video-EEG recording demonstrated a close relationship between the focal motor impairment and a clear-cut epileptic ictal discharge involving the bilateral motor cortical areas. The SPECT result was statistically analyzed by comparing with standard SPECT images obtained from our institute (easy Z-score imaging system; eZIS). eZIS revealed hypoperfusion in cingulate cortex, basal ganglia and hyperperfusion in frontal cortex,. A neuropsychological test battery revealed lower than normal scores for executive function, attention, and memory, consistent with frontal lobe dysfunction.

**Conclusion:**

The fire-setting behavior and Todd’s paralysis, together with an unremarkable performance on tests measuring executive function fifteen months prior, suggested a causal relationship between this organic brain lesion and the fire-setting behavior. The case describes a rare and as yet unreported association between random, impulse-driven fire-setting behavior and damage to the brain and suggests a disconnection of frontal lobe structures as a possible pathogenic mechanism.

## Background

The association between fire-setting behavior and psychiatric or medical disorders remains poorly understood. Thus far, studies on the characteristics of fire-setters have revealed associations with substance use disorders, personality disorders, mental retardation, schizophrenia-, mood-, and neurotic-spectrum disorders, and impulse-control disorders, including pyromania [1]. Rare associations of fire-setting behavior with sexual disorders (e.g. paraphilias), luteal phase dysphoric disorder, Asperger’s syndrome, delirious states, dementia, epilepsy, Klinefelter syndrome, XYY karyotype, Kleine–Levin syndrome, Fahr disease, and mutations in the gene encoding for monoamine oxidase A have also been reported [1-5]. Although a link between fire-setting behavior and various organic brain disorders has been established [6], associations between fire setting and focal brain lesions have not yet been reported. Transient paresis of an arm or leg following an epileptic seizure is probably the most classic lateralizing postictal paresis (PP) [7]. Here, we describe the case of a 24-year-old first time arsonist who suffered Todd’s paralysis prior to the onset of a bizarre and random fire-setting behavior.

## Case presentation

A 24-year-old, right-handed Japanese man with a ninth-grade education was referred to us by the court for pre-trial evaluation. His sister, aunt and cousin have been suffering from epilepsy. He had no history of antisocial behavior. Fifteen months ago, he suffered from headache, nausea, vomiting, general malaise, abdominal discomfort, vertigo, and tinnitus during a shopping experience and could not maintain an upright stance or walk. He was transported to the hospital, and was diagnosed with vertigo and generalized anxiety disorder. Fourteen months ago he showed the same symptoms, as well as a loss of consciousness and numbness in the legs during a lunch break. Thirteen months ago, he stopped driving because he complained about headaches and vertigo, although he could move his legs. He could not remember this episode. Eight months ago a psychiatrist diagnosed epilepsy with an electroencephalogram (EEG) and started to administer clonazepam (1 mg). After this he spent most of his days fishing without a job.

The suspect had been arrested on felony arson charges after starting at least five separate fires in residential neighborhoods in less than a month. The suspect, who had no prior arrests, indictments, or convictions, confessed to setting fires indiscriminately and seemingly without purpose to a bamboo broom, styrofoam, a towel, and a wooden board near a junior high school, a warehouse, a boat, and a shrine, respectively. When he set fire to some items he felt vertigo and could not maintain power in the legs.

The pre-trial evaluation involved the assessment of (i) the examinee’s current mental state, (ii) the examinee’s criminal responsibility, primarily depending on the examinee’s mental state at the time of the offence, (iii) the examinee’s risk for criminal recidivism, and (iv) the suitability of a court order for mental health treatment to reduce criminal recidivism.

Upon presentation, the examinee appeared to feel well but complained of difficulties concentrating and of a recent impairment in memory. He claimed to have had no intent to violate the law and appeared to have no explanation for his fire-setting behavior. He recalled waking up in the early morning hours and leaving his home to roam the residential neighborhood. He also recalled feeling somehow strange before setting fire to various items haphazardly. The examinee denied any sexual fantasies or arousal associated with the fire-setting behavior.

### Medical history

The examinee was taking no regular medication but had a history of alcohol abuse since his adolescent years. Alcohol intoxication was therefore considered as a possible cause for the fire-setting behavior but was later dismissed as no evidence for recent alcohol consumption emerged.

### Mental status examination

Initial multiaxial diagnostic assessment according to classifications provided by the Fourth Edition of the Diagnostic and Statistical Manual of Mental Disorders-Text Revision (DSM-IV-TR, American Psychiatric Association, Washington DC, 2000) revealed mild mental retardation. There was no evidence for delusions, hallucinations, manic episodes, or possible axis II disorders relevant to the fire-setting behavior. Axis III evaluation revealed a history of Todd’s paralysis, which raised the possibility of a delirious state as a possible cause for the observed fire-setting behavior [4]. Axis IV evaluation did not reveal burdening psychosocial and environmental factors including occupational and housing problems. Further investigations showed that, prior to the fire-setting, he was unemployed for eight months after a security guard job. Finally, axis V assessment of global functioning revealed a score below 65 (maximum score 100).

### Laboratory data

Laboratory results were normal or borderline pathologic. A diminished lymphocyte count (990/ml), a slightly elevated C-reactive protein (2.3 mg/l), and marginally diminished Ca^++^ (10.4 mg/l) were found. Serologic testing showed no evidence for infection with *Treponemapallidum*, *Borreliaburgdorferi*, hepatitis B or C viruses, or HIV 1/2.

### Neuroimaging data

**Figure 1:**
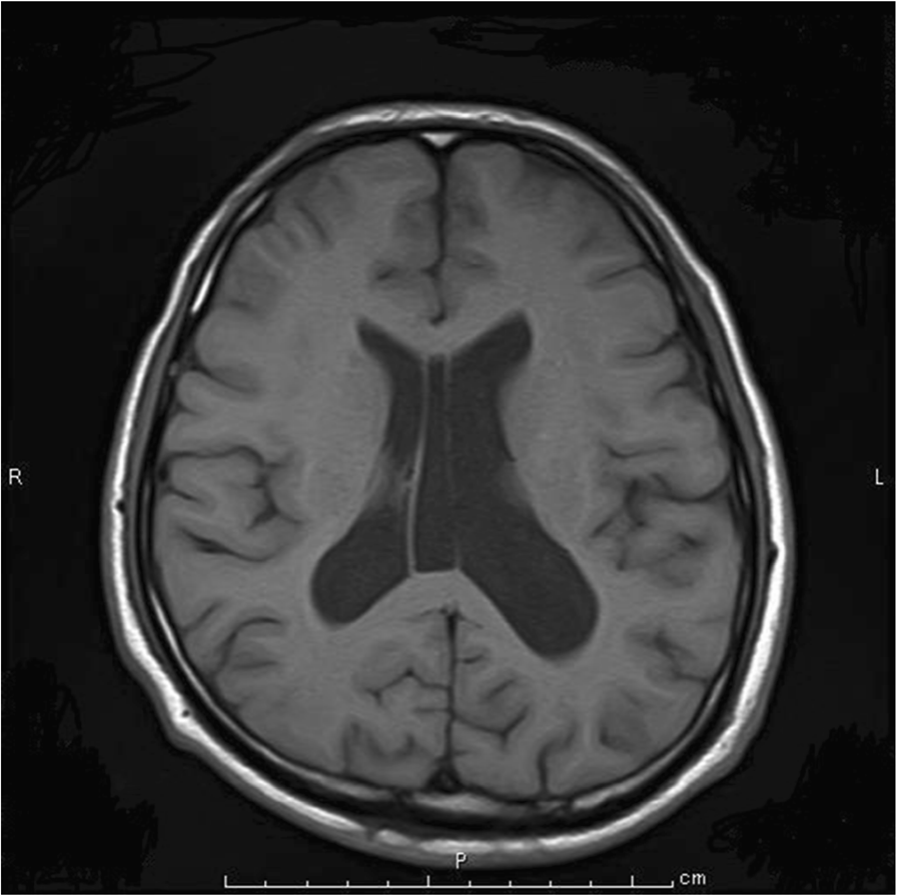
MRI of the brain and dilated lateral ventricle.

**Figure 2:**
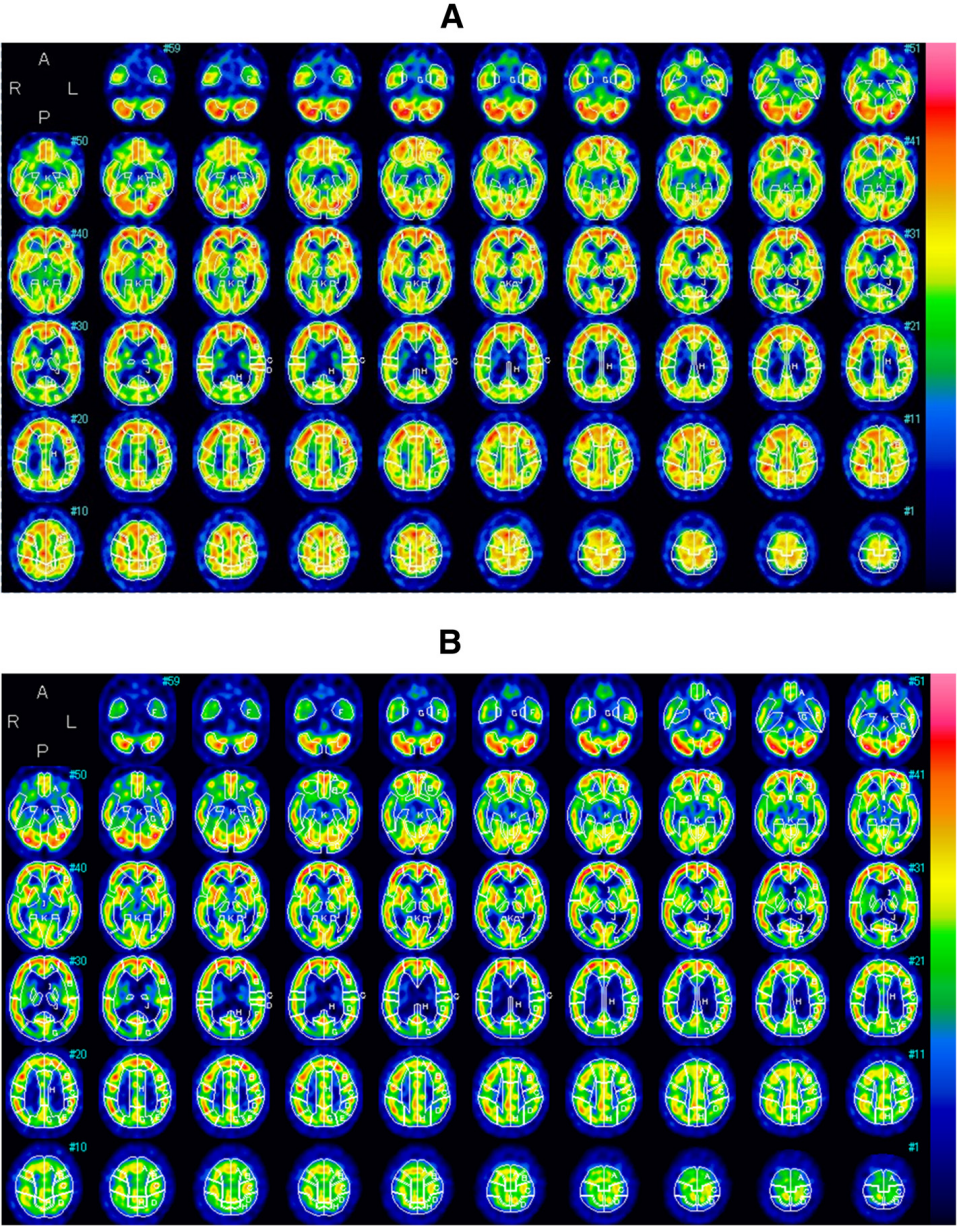
eZIS from ictal and interictal Tc-99 m ECD-SPECT (A), (B).

**Figure 3:**
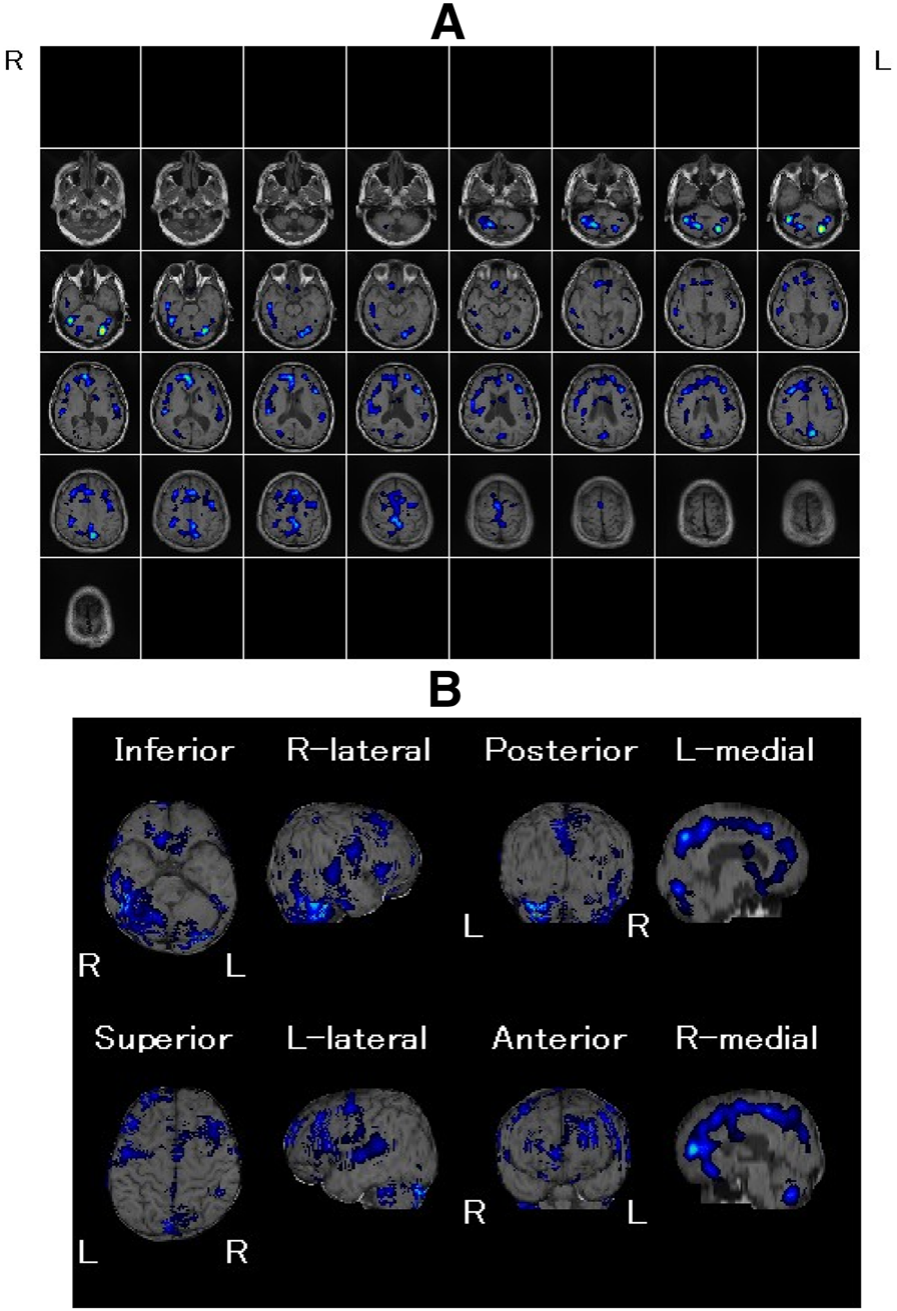
**eZIS from ictal - interictal Tc-99 m ECD-SPECT (A), (B).** This figure shows hyperperfused areas compared with the standard database. Hyperperfusion is shown as a blue to yellow area in the frontal cortex. This figure is typical of the “frontal type” of eZIS pattern.

**Figure 4:**
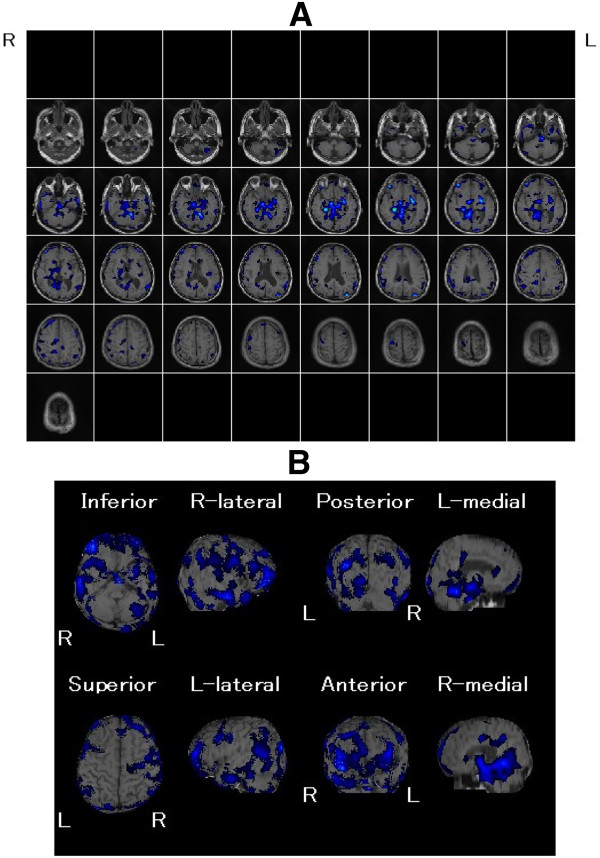
**eZIS from interictal - ictal Tc-99 m ECD-SPECT (A), (B).** Hypoperfusion is shown as a blue area in cingulate cortex, basal ganglia.

Cerebral magnetic resonance imaging (MRI) revealed atrophy of the brain and dilated lateral ventricle (Figure 1). An easy Z-score imaging system (eZIS) program was used to analyze perfusion single-photon emission computed tomography (SPECT) images of the study participants [8-10]. SPECT images obtained from each participant were subjected to anatomic standardization using statistical parametric mapping two with an original Tc-99 m ECD template followed by isotropic 12- mm smoothing and compared with the mean and standard deviation (SD) of SPECT images obtained from a normal database that had been previously established at our institute. The SPECT result was statistically analyzed by comparing with standard SPECT images obtained from our institute (easy Z-score imaging system; eZIS). eZIS revealed hyperperfusion in frontal cortex (Figure 2). eZIS from ictal – interictal ECD-SPECT showed hyperperfusion in the frontal cortex (Figure 3). eZIS from interictal – ictal Tc-99 m ECD-SPECT showed hypoperfusion in cingulate cortex, basal ganglia (Figure 4).

### Neurologic examination

Bilateral PP of the bilateral legs (motor deficits without sensory deficits) lasted approximately 5 minutes. He was confused during ictal events, and suffered from headache, and retrograde and anterograde amnesia after ictal events.

### Other diagnostic procedures

**Figure 5:**
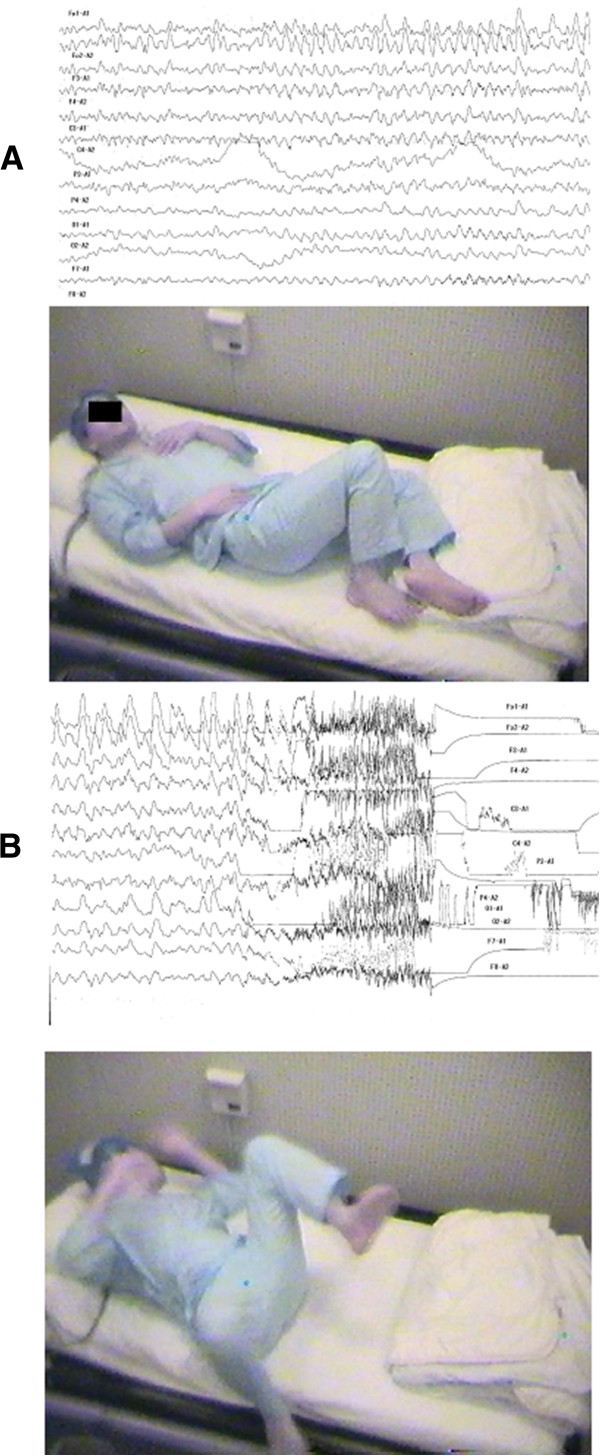
The electroclinical features of an episode of fluctuating left upper limb motor deficit that occurred during a video-EEG recording.

Further examination showed that an electrocardiogram was normal. A video-EEG was performed. The tracing just before epilepsy was characterized by the presence of irregular slow waves (Figure 5A). The tracing during epilepsy was characterized by the presence of an ictal discharge of repetitive spikes localized in the frontal, central, and parietal regions. The appearance of the motor deficit (starting from the hand) was accompanied by a clear-cut ictal discharge of repetitive spikes involving the right frontal, central, and parietal regions (Figure 5B). The discharge clearly correlated with the severity of the neurologic deficit, being more sustained when the paralysis was complete and more irregular and fragmented when it was less severe.

### Neuropsychological testing

We performed a number of neuropsychological tests with particular emphasis on the assessment of executive function and control [11]. Performance on the n-back task, which is sensitive to frontal lobe damage [12], was poor (trials correct; 1-back: 75%, 2-back: 42%, 3-back: 16%). Assessment of letter-based word retrieval using the phonemic version of the Thurstone Word Fluency Test yielded remarkably poor results (21 words produced in 3 min), consistent with a loss-of-function of the left frontal lobe [13]. With respect to the Wisconsin Card Sorting Test (WCST), the error rate, the number of categories achieved, and the number of perseverative errors of the Nelson type were 52.6%, 4, and 20, respectively. The Wechsler Adult Intelligence Scale-III (WAIS-III) revealed scores below the normal range (scaled score 10.1 ± 0.9) for three performance subtests: arithmetic (score 3), picture completion (score 1), digit symbol coding (score 12), and symbol search (score 1). These scores indicated a moderate impairment in perceptual organization and a severe impairment in processing speed, consistent with the involvement of frontal brain regions in these tasks [14]. An important finding across all neuropsychological tests was the examinee’s unawareness of his test performance.

Most likely, these cognitive impairments developed as a result of epilepsy. In particular, performance on the three WAIS-III subtests with low scores was normal in the pre-stroke condition: picture completion (score 8), block design (score 7), and digit symbol coding (score 8). The average premorbid intelligence quotient (IQ) score, obtained at the same time, was 63 (performance score 68; verbal score 64). Treatment with carbamazepine was started and led to complete seizure control.

## Discussion

Arson is frequently associated with psychiatric morbidity and with previous non-violent or violent offences [15]. In contrast, our case suggests an association between an isolated, first-time arson offence and Todd’s paralysis. Todd’s paralysis describes focal weakness in a part of the body after a seizure. Our case showed bilateral leg weakness, and this weakness recovered completely within 48 hours. The precise mechanism that links damage of the motor cortex to this complex, impulse-driven behavior is not known. Neuropsychological and neuropsychiatric findings suggest temporal and frontal lobe dysfunctions in violent offenders and these dysfunctions appear to be more pronounced in the dominant hemisphere [16].

However, lesions in the hypoperfused regions within the frontal cortex and basal ganglia might result in a disconnection and deactivation of the frontal cortex. Consistent with this concept are more recent observations linking cognitive inflexibility with prefrontal depletion of serotonin [17]. Moreover, and in agreement with our findings, neuropsychological deficits (including memory impairment) have been associated with hypoperfusion within the frontal cortex and basal ganglia [18,19]. Finally, a neural framework controlling impulsive behavior, decision-making, and willpower has been proposed incorporating components of this framework, and is primarily localized to frontal lobe structures [20].

Here we argue that, in our case, disconnection of the frontal lobe resulted in impairments of cognition, primarily executive functions involving planning and judgment, as well as partial loss of memory and impulse control functions. Together, this led to a bizarre type of impulse-driven, non-intentional fire-setting behavior. Consequently, the examinee’s capacity to form a specific intent at the time of the offence remained uncertain, implying lack of capacity to form *mensrea*[21], which, at least in part, negated criminal responsibility.

Based on our pre-trial evaluation, a diagnosis of personality change (disinhibited behavior) due to brain damage and dysfunction (DSM-IV-TR: 310.1) was made, combined with anosognosic features (i.e., the suspect’s unawareness of his impaired cognitive functions). The examinee was found not criminally responsible. However, the examinee was deemed at risk for criminal recidivism as no specific treatment for his condition existed. We recommended continuing prophylactic treatment with antiepileptic medication to help prevent further hypoxic-ischemic insults. The case was closed with a verdict of guilty by reason of legal sanity and the examinee was transferred to a prison. Neuropsychological reassessment may help estimate the examinee’s risk for future criminal recidivism.

## Conclusion

The fire-setting behavior and Todd’s paralysis, together with an unremarkable performance on tests measuring executive function fifteen months prior, suggested a causal relationship between this organic brain lesion and the fire-setting behavior. The case describes a rare and as yet unreported association between random, impulse-driven fire-setting behavior and damage to the brain and suggests a disconnection of frontal lobe structures as a possible pathogenic mechanism.

## Abbreviations

DSM-IV-TR, The Fourth Edition of the Diagnostic and Statistical Manual of Mental Disorders, Text Revision; EEG, Electroencephalogram; eZIS, Z-score imaging system; MRI, Magnetic resonance imaging; WAIS-III, The Wechsler Adult Intelligence Scale-III; SPECT, Single-photon emission computed tomography.

## Competing interests

Authors have no competing interests to declare that are relevant to the content of this submission.

## Authors’ contributions

MK and KM followed up the patient during the admission. YM and TN participated in EEG study. YI and YT examined SPECT study, HK, JT and HH examined psychological test examination, JA planed the study, and participated in its design and coordination and helped to draft the manuscript. All authors read and approved the final manuscript.

## Pre-publication history

The pre-publication history for this paper can be accessed here:

http://www.biomedcentral.com/1471-244X/12/132/prepub
